# Emerging roles for Mitochondrial Rho GTPases in tumor biology

**DOI:** 10.1016/j.jbc.2024.107670

**Published:** 2024-08-14

**Authors:** Dillon P. Boulton, M. Cecilia Caino

**Affiliations:** 1 Department of Pharmacology, University of Colorado School of Medicine, Aurora, Colorado, USA; 2 Pharmacology Graduate Program, University of Colorado, Aurora, Colorado, USA; 3 University of Colorado Cancer Center, University of Colorado Anschutz Medical Campus, Aurora, Colorado, USA

**Keywords:** MIRO1, MIRO2, mitochondria, cancer, metastasis

## Abstract

Mitochondrial Rho GTPases (MIRO1 and MIRO2) are primarily studied for their role as resident mitochondrial anchor proteins that facilitate mitochondria trafficking in neurons. However, it is now appreciated that these proteins have critical roles in cancer. In this review, we focus on examining the role of MIROs in cancer, including expression changes in tumors and the molecular mechanisms by which MIROs impact tumor cell growth, invasion, and metastasis. Additionally, we give an overview of how MIRO’s functions in normal cells within the tumor microenvironment can support or inhibit tumor growth and metastasis. Although this is still an emerging field, the current consensus is that the MIROs primarily promote tumor progression of disparate tumor types. As mitochondrial proteins are now being targeted in the clinic, we discuss their potential as novel proteins to target in cancer.

While mitochondria are best known for their role in ATP production, they are now recognized as highly dynamic organelles with important functions in apoptosis, calcium buffering, redox homeostasis, biosynthesis, and cell signaling (1, 2, 3, 4). As a part of their dynamic nature, mitochondria change their shape, size, organelle–organelle contacts, metabolism, and localization to support cell function and adapt to extracellular and intracellular cues (5). Unsurprisingly, disruption in mitochondrial dynamics has been observed in a variety of pathologies including neurological disorders, cardiovascular disease, and cancer (6, 7, 8). The core machinery responsible for organelle shape and localization is conserved among normal and cancer cells, and alterations in the expression and/or regulation of the key molecular players that orchestrate mitochondrial shape changes have been linked to tumor progression (9, 10).

In this context, outer mitochondrial membrane proteins—Mitochondrial Rho GTPases (MIRO1/2, also known as RHOT1/2)—are a family of atypical GTPases that have demonstrated functions in the regulation of mitochondrial function and mitochondrial dynamics. MIRO1 and MIRO2 share 60% sequence homology and several unique features that differentiate them from other small monomeric GTPases (11). MIROs have two distinct GTPase domains that are separated by two calcium binding EF hand motifs (11) and are stably anchored in the mitochondrial membrane *via* a hydrophobic transmembrane domain (12). Initial studies noted a change in the mitochondrial network of COS-7 cells upon transient overexpression of GTPase domains mutants of MIROs (12). Due to these observations, a majority of the field has focused on the role of MIROs in mitochondrial trafficking, where MIROs connect to kinesin and dynein motors through the TRAK1/2 adapter proteins (13, 14). As the role of MIROs continues to be studied outside of neurons, the consensus is that MIRO1 has a highly conserved function for mitochondrial trafficking in many mammalian cell types spanning immune, mesenchymal, and epithelial cells of normal and diseased origins (15, 16, 17). In contrast, MIRO2 seems to play a far less critical role, with several studies observing complete dispensability for MIRO2 in mitochondrial trafficking under basal conditions (16, 18). Elegant studies exploring overlapping roles of MIROs have suggested that MIRO2 likely retains the ability to mediate mitochondrial trafficking; however, MIRO1 acts as the primary regulator of this process (16). In support of this concept, in mouse embryonic fibroblasts (MEFs), MIRO1 can fully compensate for ablation of MIRO2, while MIRO2 is only able to minimally compensate for loss of MIRO1 (16). Furthermore, MIRO2 is required for mitochondrial trafficking in the context of adaptive resistance to PI3K inhibitors in glioblastoma cells (19). It is possible that MIRO2 acts as a “back-up” for MIRO1 in instances of cellular stress and is not required under normal conditions.

More recent studies show a variety of alternate functions for MIROs outside of mitochondrial trafficking, including regulation of mitochondrial shape (12), cristae formation (20), connection of mitochondria-ER membranes (20), reactive oxygen species production (21), promotion of mitochondrial-derived vesicle formation (22) and mitophagy (23) (Fig. 1). Additionally, newer evidence highlights non-mitochondrial functions for MIROs, as two out of four MIRO1 isoforms are partially localized to peroxisomes (24) to permit short-range peroxisomal trafficking and inhibit peroxisomal fission in collaboration with a peroxisome-localized pool of MIRO2 (25). Thus, MIROs have emerged as important regulators of organelle dynamics in mammalian cells, playing crucial roles in pathophysiological development of many diseases. In particular, MIRO1 has been implicated in many neurological disorders and is essential for proper neurodevelopment in mammals (26). The roles of MIROs in neurobiology and beyond have been reviewed elsewhere (27, 28, 29). Here, we focus on examining the role of MIROs in cancer, as MIROs are emerging as critical regulators of tumor biology. In this review, we will discuss the evidence that indicates key roles for MIRO1 and MIRO2 in the function of both the tumor cells as well as the cells in the surrounding microenvironment of various tissues that impact tumor growth and progression.

**Figure 1:**
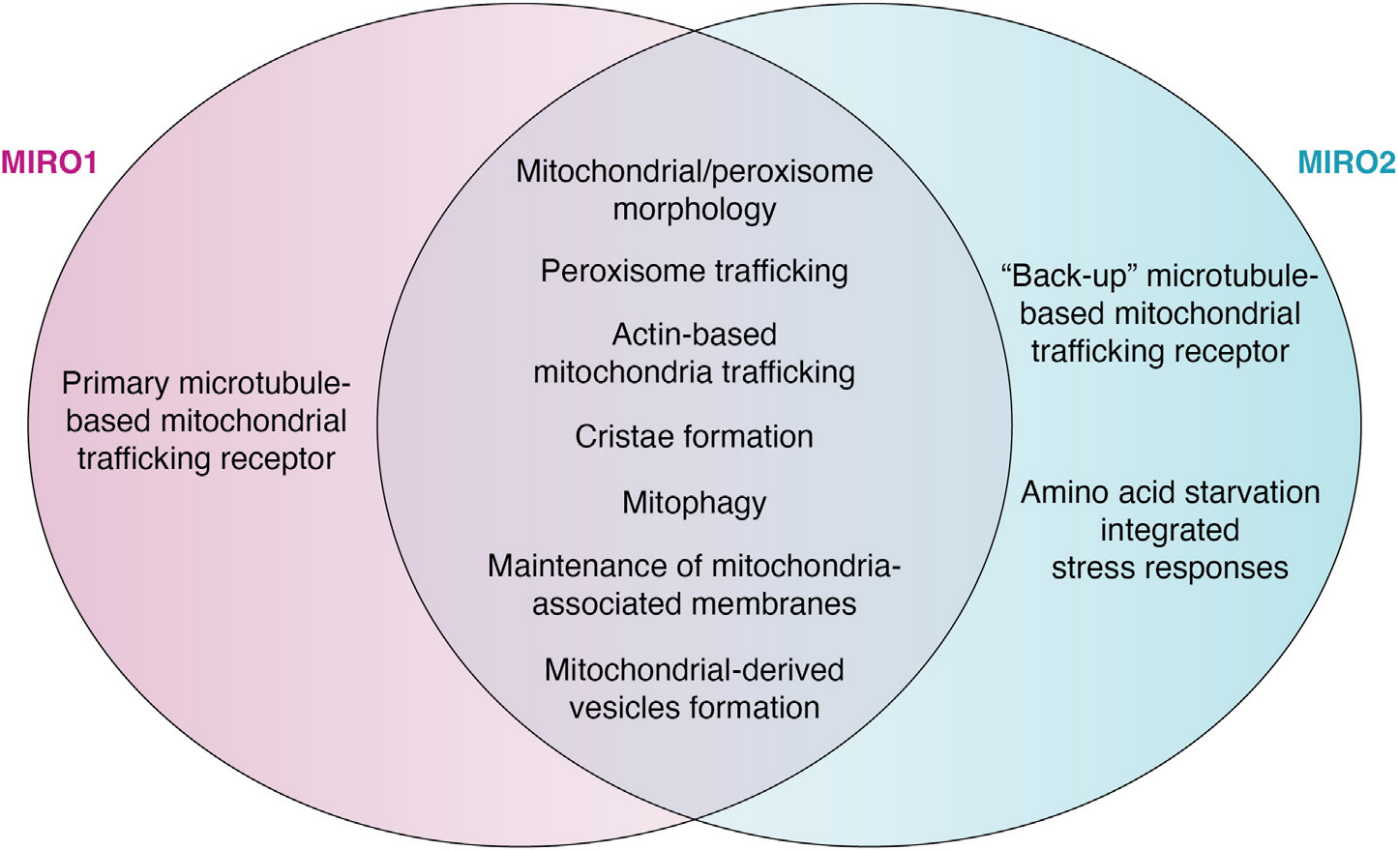
**Overlapping and unique molecular functions of MIROs.** An overview of the many different overlapping and nonoverlapping functions of the MIROs. MIRO, mitochondrial Rho GTPase.

## MIRO expression, genomic alterations, and regulation in cancer

Several studies examined the expression of MIRO1 in cancer *versus* normal tissues, with disparate results depending on the tumor type. MIRO1 has increased expression in tumors compared to adjacent normal tissue in hepatocellular carcinoma (HCC) (30), gastric cancer (31), and pancreatic cancer (PC) (32). However, there is some controversy as one report found MIRO1 expression is decreased in the tumor compartment, with higher expression being associated with better outcomes for patients (33). The differences between the two studies in PC could be explained from the utilization of different PC patient cohorts. Additionally, other studies found no differences in *MIRO1* expression between normal and bulk tumor tissue in HCC, breast cancer (BCa), and ovarian cancer (21). Interestingly, within these same tumor types, *MIRO1* was increased in cancer-associated fibroblasts (CAFs) compared to normal fibroblasts, highlighting changes in *MIRO1* expression associated with the stromal cell compartment of tumors.

Adding to the evidence that many tumors have altered expression of MIRO1 expression, there is an abundance of work that describe the importance of MIRO1 circular RNA in tumors. Circular RNAs are derived from alternatively spliced pre-mRNA and are now emerging as another form of noncoding RNA that are hijacked by cancer cells (34). Circular MIRO1 RNA (circMIRO1/circRHOT1) is increased in many different tumor types including, non-small cell lung cancer (NSCLC) (35), HCC (36), BCa (37), and PC (38). Whether cirMIRO1 and *MIRO1* expression in tumor tissues is correlated is rarely examined. One study using HCC patient samples showed no correlation between circMIRO1 and *MIRO1* expression (36), suggesting that the *MIRO1* mRNA and circRNA may be differentially regulated.

A few reports examined the expression of *MIRO2* in cancer *versus* normal tissues. In a pan-cancer analysis of microarray and RNAseq public databases, we found that *MIRO2* expression was increased in tumor compared to normal tissues in most tumor types, correlating with worse patient outcomes in tumors of the breast, kidney, lung, and skin cutaneous melanoma (19). Unfortunately, very few studies have directly compared the expression of *MIRO1* and *MIRO2* in tumor tissues. In a recent analysis of The Cancer Genome Atlas Program Pan Cancer study dataset, we reported that median *MIRO2* expression trended higher than *MIRO1* expression within the same tumors, across all of the tumor types (18). In prostate cancer (PCa), we found that *MIRO1* and *MIRO2* mRNA expression were oppositely regulated in tumor *versus* normal tissue (18). While PCa patients tended to have higher *MIRO2* expression in tumor *versus* normal tissue, *MIRO1* expression was typically lower in the tumor tissue in comparison to the normal tissue. In this context, higher *MIRO2* expression correlated with worse disease-free survival in patients, while *MIRO1* expression showed no correlation with patient outcomes.

The status of genomic alterations and mutations for MIRO2 have been addressed in a single study. In The Cancer Genome Atlas Program database, the most common genomic alteration for MIRO2 was amplification (up to 20% of pancreatic tumors, 15% of prostate adenocarcinoma and neuroendocrine prostate tumors, and under 10% in breast tumors) (18). Mutation of MIRO2 was the second most frequent alteration seen in select datasets, including cervical and uterine carcinomas and prostate organoids. These mutations were randomly distributed throughout the functional domains of MIRO2, with a few recurrent point mutations with low frequency (3–5 cases) associated with functional domains. A subset of MIRO2 mutations found in PCa patients were analyzed for their binding to a cancer-specific binding partner, general control of non-derepressible 1 (GCN1). At the molecular level, these mutants (MIRO2 159L) increased GCN1 binding, and MIRO2 277R decreased GCN1 binding (18); however, a potential effect of expression of these MIRO2 mutants in regulating tumor cell-intrinsic phenotypes was not explored. Furthermore, the majority of patient-associated MIRO2 mutations have not been characterized in terms of functional relevance yet. A similar analysis investigating potential genomic alterations for MIRO1 has not been reported.

Despite the abundance of evidence demonstrating altered MIRO1 or MIRO2 expression in many tumors, how either MIROs expression are regulated remains poorly understood. Several recent reports have identified chromosomal proteins and transcription factors involved in the regulation of MIRO1 and MIRO2 in cancer. In HCC, HMGB1—a chromosomal protein known to alter chromatin structure and regulate transcription (39)—is sufficient to promote MIRO1 expression and is necessary for hypoxia-dependent increases in MIRO1 (30). In CAFs, MIRO1 expression was stimulated through a signaling cascade involving Activin A and SMAD2/3 (21). It is unknown whether these expression changes through these transcription promoting factors is exclusive to MIRO1 or also affects MIRO2 expression. Addressing this, a study showed that MYC amplification drives increased expression of many proteins involved in mitochondrial trafficking, including MIRO1 and MIRO2 (17). MYC is one of the most frequently amplified genes in cancer and has been extensively studied for its role as a transcription factor to promote tumorigenesis (40, 41). It is possible that increases in either MIROs expression in so many disparate tumor types is—at least in part—from the broad effects of MYC amplifications in these tumors. Interestingly, expression of MYC was shown to be partially controlled by circMIRO1 (35). We speculate that this may provide a feedforward loop where MYC could promote transcription of MIRO1 and circMIRO1, which then stimulates MYC expression.

Post-translational mechanisms may also contribute to regulation protein levels of MIROs in cancer. For instance, actin dynamics regulator—mDia2—protects MIRO1 from proteasomal degradation in a manner that is dependent upon mDia2’s ability to polymerize actin in CAFs (21). While studied in the context of nontumorigenic cells, MIROs have also been shown to be important for efficient lysosomal-dependent degradation of mitochondria, termed mitophagy (23, 42, 43). Indeed, MIROs are phosphorylated and ubiquitinated, respectively, by PINK1 and Parkin which serve as one of the main canonical mediators of stress-induced mitophagy (44, 45, 46). Interestingly, while MIRO1 is degraded upon stimulation of mitophagy, MIRO2 shows a delayed onset of degradation or does not get degraded (42, 44, 47). Furthermore, MIRO1 degradation during mitophagy may be pathologically relevant as resistance to mitophagy and subsequent protection of MIRO1 degradation has been observed in patients with Parkinson’s disease (48). However, it is unknown whether mitophagy and subsequent degradation of MIROs occurs in transformed tumorigenic cells and what the relative contribution to maintaining MIROs expression is in the context of cancer.

In sum, most reports find that circMIRO1 and MIRO2 expression are increased in tumors with MIRO1 having different patterns of expression dependent on the tumor type. Future work will need to center around understanding how these changes are regulated in tumor cell intrinsically and whether the same factors regulate MIRO1 and MIRO2 together or individually. Furthermore, it will be important to determine the effects of the tumor microenvironment (TME) on driving expression changes. As an example of this, hypoxia has been shown to induce the expression of MIRO1 in HCC cell lines (30), and *MIRO2* expression correlated with the Buffa Hypoxia score in patients with PCa (18).

## Tumor cell-intrinsic functions of MIROs

### MIRO1

Mitochondrial trafficking to the periphery of the cell is crucial for efficient cell migration (49) and is an emerging mechanism that cancer cells hijack to metastasize, reviewed in depth here (50). Indeed, mitochondria are actively transported to the leading edge of migrating cells (51) and are primarily localized toward the front end of moving cells (52). It is proposed that mitochondrial redistribution supports cell movement by regionally supplying energy (ATP and GTP) and signaling cues such as calcium and reactive oxygen species to support molecular processes occurring during cell migration (49, 53). In this vein, MIRO1 influences ATP gradients, effectively reducing the amount of ATP at the cell periphery in MEFs (53) and CAFs (21). Furthermore, MIRO1 enables H_2_O_2_ production at the leading edge, which in turn modulates focal adhesion dynamics in MEFs (54).

**Figure 2:**
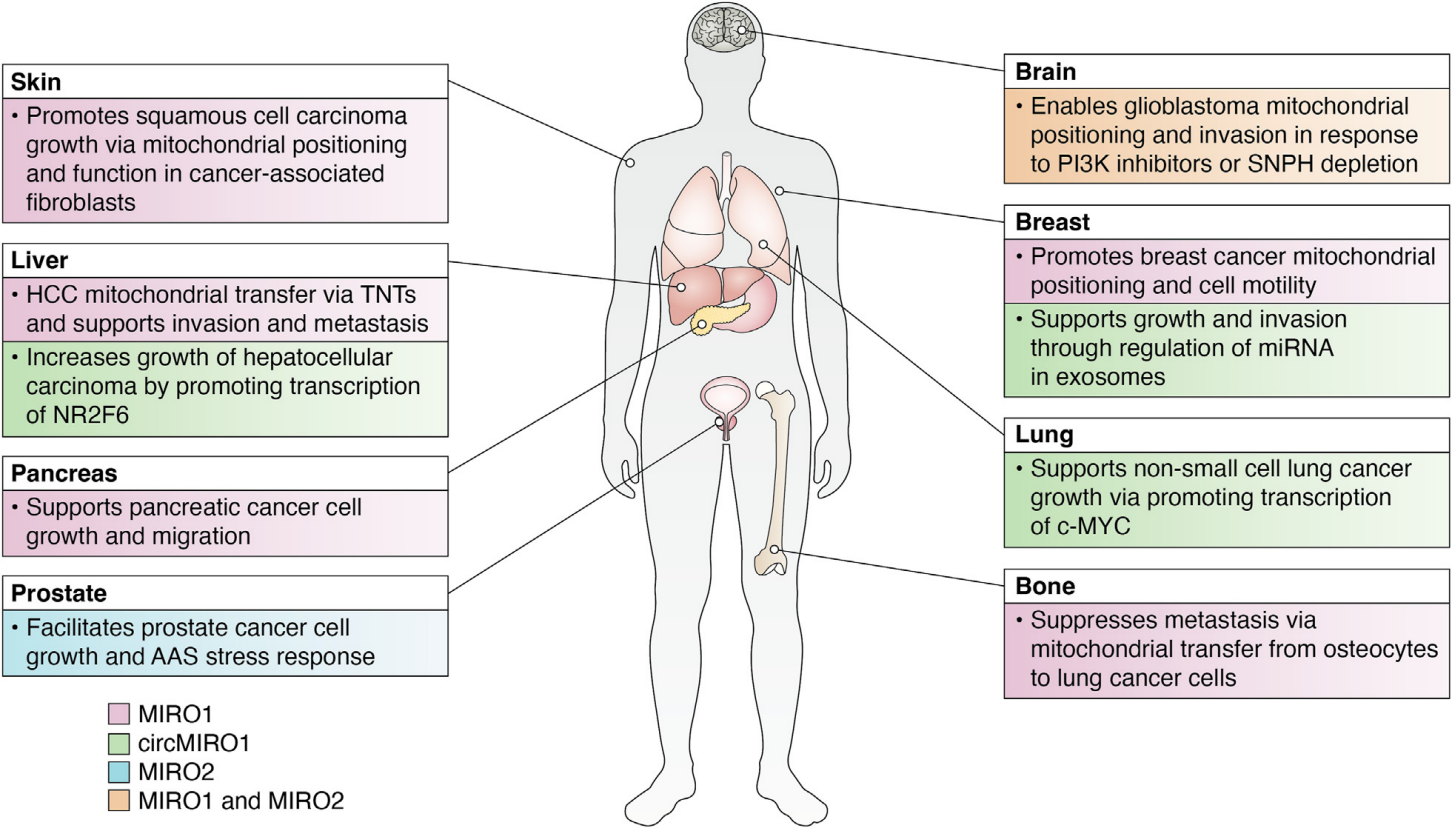
**Functions of MIRO1, circMIRO1, and MIRO2 in different tumors and tissues.** An overview of the reported functions of MIROs in disparate tumor types. AAS, amino acid starvation; HCC, hepatocellular carcinoma; MIRO, mitochondrial Rho GTPase; TNT, tunneling nanotubes; SNPH, Syntaphilin. Created in BioRender.com.

Based on the role of MIRO1 in strategically positioning mitochondria to support cell motility, it is not fully surprising that several studies have found MIRO1 to be important in cancer cell migration and invasion (Fig. 2). Using BCa cell models, one study showed that depletion of MIRO1 disrupted mitochondrial localization to the anterior regions of cells during cell migration and was concurrent with a reduction in cell velocity and persistence (52). Furthermore, we have found that depletion of the mitochondrial anchoring protein Syntaphilin resulted in dramatic redistribution of mitochondria toward the cortical region of cancer cells and led to exacerbation of tumor cell motility in PCa cell models (19). In addition, exacerbated mitochondrial accumulation in the cortical region was observed as adaptive response to targeted therapies, including quick metabolic and signaling reprogramming in response to PI3K inhibitors in PCa and glioblastoma cells models (49). Cortical mitochondrial localization and tumor cell motility were fully dependent on MIRO1 in both contexts.

In addition to linking mitochondria to microtubules, MIRO1 has been shown to mediate transfer of mitochondria between cells *via* nanotubes (55). A recent study demonstrated that transfer of mitochondria from highly invasive HCC cells—through tunneling nanotubes—was sufficient to increase invasion of low invasive HCC cells (30). In this context, MIRO1 promoted mitochondrial transfer between HCC cells as well as cell migration, invasion, and late-stage metastasis. However, it remains unclear whether MIRO1’s impact on invasion and metastasis is through mitochondrial transfer between adjacent cancer cells or due to the loss of mitochondrial trafficking within individual cells. Finally, MIRO1 was demonstrated to be important in PC cell migration and growth (32). Mechanistically, ablation of MIRO1 expression increased SMAD4 expression. SMAD4 is thought to be a tumor suppressor with mutation or deletion of SMAD4 correlating with a worse survival rate for patients with PC (56, 57). However, it is unknown whether MIRO1-dependent growth and migration are dependent on suppression of SMAD4 in this context. Overall, MIRO1 seems to support cell motility, invasion, and metastasis through its ability to transport mitochondria from perinuclear to cortical regions within the cell. It is interesting to note that MIRO1-dependent cell movements are often ascribed to MIRO1’s ability to promote mitochondrial motility without consideration to the multitude of other impacts on mitochondrial function that have recently been described. Moving forward it will be important to disentangle how the alternate functions of MIRO1 contribute to these cellular phenotypes.

### circMIRO1

As described above, many studies identified increased circMIRO1 expression in tumors as opposed to normal adjacent cells. Circular RNAs add another layer of regulation for protein expression by promoting gene expression and acting as molecular sponges for microRNAs (miRNA) (34). Several reports indicate that circMIRO1 binds to multiple different miRNAs to regulate cell growth, migration, invasion, and tumor growth *in vivo* (37, 38, 58, 59). Interestingly, circMIRO1 was also found to be increased in both the tumor and exosomes of BCa patients (37). Here, exosomes from a BCa epithelial cell line (MCF-7) were able to stimulate growth, migration, and invasion to greater extent than exosomes from normal breast epithelia (MCF-10A), in a manner that was dependent on circMIRO1. Mechanistically, circMIRO1 blocked miR-204-5p degradation of protein arginine methyltransferase—PRMT5—which was responsible for promoting growth, migration, and invasion.

A second mechanism by which circMIRO1 regulates growth, migration, and invasion through recruitment of histone acetyltransferase, KAT5 (35, 36), has also been reported. In NSCLC cells, circMIRO1-mediated c-MYC expression through recruitment of KAT5 and subsequent H3K27ac and recruitment of RNA pol II to c-MYC promoter regions (35). In this context, exogenous expression of c-MYC in the absence of circMIRO1 only partially recovered tumor growth *in vivo*, suggesting that regulation of c-MYC expression may be only one of many mechanisms circMIRO1 promotes tumor growth. Indeed, circMIRO1 was also found to be important for growth, migration, invasion, and tumor growth *in vivo* in HCC (36). Performing RNA-seq on HCC cells, it was found that NR2F6 was the most decreased transcript in cells depleted of circMIRO1. NR2F6 is an orphan nuclear factor that functions as a tumor promoting protein in both tumor and immune cells (60). In the context of HCC, circMIRO1 mechanistically controlled NR2F6 expression by increasing chromosomal accessibility and recruitment of KAT5 and RNA pol II to promoter regions surrounding NR2F6 (36). Re-expression of NR2F6 in the absence of circMIRO1 rescued growth, migration, invasion, and tumor growth *in vivo*. It is interesting to note that while exogenous expression of NR2F6 following circMIRO1 depletion in HCC xenograft models fully rescued tumor growth, exogenous expression of c-MYC had only a partial rescue of tumor growth in NSCLC xenograft models.

### MIRO2

The literature exploring MIRO2’s role in the context of tumor cell biology is limited to a few contributions. The first report examining MIRO2 in cancer showed that MIRO2 depletion partially reduced tumor cell invasion in PCa cells; however, MIRO2 was dispensable for exacerbated tumor cell invasion induced by depletion of Syntaphilin (19) (Fig. 2). A second study examining mechanisms of tumor cell motility and invasion downstream of MYC identified MIRO2 as a target of MYC (17). Of note, MIRO1 and kinesins were also co-regulated by MYC, contributing to exacerbated mitochondrial trafficking downstream of MYC. In this context, restoring MIRO2 expression in MYC-depleted cells led to rescue of cortical mitochondria and PCa tumor cell motility and invasion. More recently, we showed that MIRO2 was necessary for 2D cell growth, anchorage-dependent growth, and anchorage-independent growth in a panel of PCa cell lines (18). Furthermore, MIRO2 depletion reduced PCa xenograft *in vivo*, which was associated with a decrease in proliferative index without affecting cell death. In these studies, MIRO2 depletion did not affect subcellular distribution of mitochondria to the cortical region of PCa cells, suggesting that MIRO2-dependent growth relied on alternative mechanisms. This is in agreement with evidence that mitochondria do not primarily rely on MIRO2 for long-range mitochondrial distribution under basal conditions (16). However, in MEFs, MIRO2 cooperates with MIRO1 to mediate shorter distance mitochondrial movements on actin filaments *via* its interaction with and stabilization of motor protein MYO19 (16). In this context, both MIROs seem to contribute to proper segmentation of mitochondria during cell division, supporting efficient cell mitosis. This may serve as an important function for both MIROs in cancer cell growth, although this possibility remains to be tested.

In terms of mechanisms that explain MIRO2-depedent tumor growth, we demonstrated that MIRO2 serves as a key component of a unique signaling node. Using an unbiased proteomics screen, we identified a large cohort novel binding partners for MIRO2 including GCN1 (18). GCN1 is the co-activator of the ser/thr kinase GCN2/EIF2AK4—one of four major kinases involved in stress sensing in the integrated stress response (ISR). GCN2 senses stress through a variety of intracellular cues including amino acid availability, oxidative stress, and actin dynamics cues (61, 62). We found that MIRO2 was critical for cell growth by mediating efficient ISR signaling—including phosphorylation of eiF2⍺ and inducing ATF4 expression—in response to amino acid starvation (18). Interestingly, MIRO1 had seemingly divergent functions from MIRO2 in this context as ablation of MIRO1 did not impact cell growth or ISR signaling. At the molecular level, although GCN1 co-precipitated with GTP-bound MIRO2, the MIRO2-GCN1 complex was not affected by MIRO2’s GTPase active/inactive conformation. Indeed, GCN1 bound to a similar extent to MIRO2 bearing N-terminal and/or C-terminal GTPase active (13V or 425V) or inactive (18N or 430N) mutants. Furthermore, double N-terminal and C-terminal GTPase mutations representing all potential combinations yielded similar levels of GCN1 binding to these MIRO2 mutants (18).

Taken together, we find that MIRO1, circMIRO1, and MIRO2 have important functions in tumor cell-intrinsic phenotypes, including promoting tumor growth, cell motility, and metastasis. While MIRO1 seems to function primarily through promoting mitochondrial trafficking, circMIRO1 influences many of these phenotypes by regulating mRNA and protein expression, through promoting transcription and repression of miRNA expression. Excitingly, our recent study also indicates that the MIRO2 may be acting as a signaling platform to promote efficient signaling of stress response pathways. Work in the future will need to center around whether MIRO2 is important exclusively for tumor growth or whether it is important throughout tumor progression. Furthermore, our identification of many novel MIRO2 binding partners (18) suggests a variety of unknown functions and signaling pathways in which MIRO2 is involved and will need to be pursued in the future. Overall, these reports demonstrate the multifaceted roles for the MIROs in cancer and underscore their importance as potential therapeutic targets.

## Tumor cell-extrinsic functions of MIROs

It is now highly appreciated that the unique milieu of normal cells, cancer cells, and extracellular factors that exist within a tumor—termed the TME—are extraordinarily important in tumorigenesis and tumor progression (63, 64). In this context, two recent studies underscore the importance of MIRO1 in the TME. One group demonstrated that MIRO1 plays a protumor role in CAFs, which in turn promoted squamous cell carcinoma growth, invasion, and tumor growth *in vivo* (21) (Fig. 2). MIRO1 was protected from proteasomal degradation by binding to mDia2—a novel binding partner identified in this study—and was required for proper mitochondrial function and localization in CAFs. Similar to what was found in MEFs (53), MIRO1 was responsible for regional ATP supply at the cell periphery in CAFs and was necessary for the secretion of pro-tumorigenic proteins.

A different study argues for an anti-metastatic role of MIRO1 *via* osteocyte transfer of mitochondria to lung cancer cells (65). Utilizing an osteocyte conditional MIRO1 knockout (MIRO1^CKO^) mouse model, MIRO1^CKO^ mice developed larger bone metastases, concurrent with fewer mitochondria transferred from osteocytes to the cancer cells. This is contrast to HCC cells which found mitochondrial transfer between highly to lowly invasive cells increased their invasive capacity (30), suggesting MIRO1-dependent mitochondrial transfer may be pro-metastatic or anti-metastatic, depending on the context and the source of mitochondria being transferred. Indeed, blocking mitochondrial transfer from osteocytes to tumor cells—*via* MIRO^CKO^—blocked an inflammatory cGAS-STING signature in tumor cells and decreased antitumor immune responses (65). Additionally, old mice had higher intra-tibial metastatic burden than young mice, with subsequent sequencing of the bone tissue showing reduced *MIRO1* expression in the bones of old mice in comparison to bones of young mice. In these studies, *MIRO2* also had reduced expression in bones of aged mice in comparison to young mice. It is interesting to speculate that MIRO2 may also modulate the metastatic potential of cancer cells in this context, either through controlling mitochondrial transfer between osteocytes and cancer cells or other alternative functions. However, there are currently no studies that have looked at how MIRO2—in normal cell types—affects tumor progression. Furthermore, how MIROs in cancer cells may reciprocally affect the TME remains unknown.

While limited in scope, early evidence suggests MIRO1 plays an important role in TME cells to either promote or inhibit tumor growth and metastasis. Moving forward, efforts will need to systematically discover additional roles of MIRO1 and MIRO2 in the TME, in regulating normal and transformed cell compartments as well as extracellular conditions. Emphasizing this importance, using single cell RNA sequencing analysis, MIRO1 was expressed at equally high levels in macrophages as CAFs (21). Indeed, MIRO1 is important in T-cell mitochondrial rearrangement, cell motility, and adhesion (15); however, whether MIRO1 influences tumorigenesis and tumor progression *via* regulation of immune cells function remains an open question.

## Discussion

Despite a generalized focus of MIRO1 and MIRO2 in the context of neurobiology, it is becoming more appreciated that these proteins play a crucial role in tumor progression and metastasis of disparate cancer types (Fig. 2). Indeed, both MIRO1 and MIRO2 have dysregulated expression in cancer, and these changes in expression have been shown to correlate with worse patient survival. Mechanistically, MIROs serve as tumor promoting proteins by permitting spatial organization of mitochondria, regulating expression of many different genes and controlling signaling pathways within cancer cells. It is also becoming clear that these proteins have important roles in dictating how normal cells in the TME interact with adjacent tumor cells and how this crosstalk ultimately impacts cancer growth.

One of the many open areas in this field centers around parsing out overlapping and non-overlapping functions of MIRO1 and MIRO2 (Fig. 1). For example, MIRO1 has a conserved role in mitochondrial trafficking in many cell types, while there is controversy around whether MIRO2 is required for this process (17, 18, 26, 66). In favor or non-overlapping functions of MIROs, we have found that MIRO2 is required for growth and mediation of GCN2-dependent stress responses in PCa, while PCa cells do not require MIRO1 to mediate these processes (18). Interestingly, several studies show that MIRO1 and MIRO2 can compensate for each other in MEFs, arguing for overlapping function of MIROs. This overlap is evident in several cellular functions, including maintenance of mitochondria to ER-membranes, proper cristae formation, MYO19 recruitment and stabilization, efficient regulation of mitosis, and peroxisomal trafficking (16, 20, 25). Unfortunately, the role of MIROs in regulating most of these cellular functions has not been studied in cancer cells and normal cells simultaneously. Thus, two possibilities arise from this conundrum. First, MIROs can compensate in normal cells, whereas in cancer cells—where the expression of these proteins is often dysregulated—MIROs are no longer able to compensate for each other. The second possibility is that MIROs may have redundant functions only in specific cellular and molecular processes. An additional layer of complexity is that MIROs may lose functionality and/or gain novel functions in the context of cancer. In support of this, our proteomics experiments in cancer cells found novel MIRO2 interacting proteins, while many canonical binding partners—identified in normal cell types—were not found in our screen (18).

A second gap in the field relates to our understanding of the regulation of MIROs protein function in cancer. MIROs have many layers of regulation with the most studied being their regulation by binding to small regulatory molecules (GTP and Ca^2+^). Indeed, despite being considered atypical GTPases MIROs have functional N- and C-terminal GTPase domains (67), of which the N-GTPase domain of MIRO1 that has been suggested to regulate mitochondrial and peroxisomal distribution (12, 13, 68). Furthermore, Ca^2+^ binding to MIRO1 EF hands has been shown to inhibit mitochondrial movement in neurons (14, 69). MIROs can also localize to both mitochondria and peroxisomes, providing a secondary layer of regulation where—at least in the case of MIRO1—alternative splicing leads to changes in subcellular localization (24, 25). In the context of the cancer studies reviewed here, we found a paucity of studies examining the requirement of the GTPase domains or the EF hand motifs of MIROs to regulate tumor cell biology. Addressing this gap may provide new insight into the molecular mechanisms by which MIROs regulate tumor cell biology and indicate whether the GTPase or calcium binding activities may be targetable in cancer. Adding to this complexity, MIROs are also post-translationally modified *via* phosphorylation and ubiquitination (44, 45, 70), altering mitochondrial motility and quality control. However, it is unknown if and how any of these regulatory mechanisms are altered in cancer and how this ultimately affects tumor cell function. It is likely that there are novel tumor-specific regulatory events, given dramatic dysregulation of cell signaling within cancer.

Finally, the emergence of literature over the past decade demonstrating MIROs as important regulators of tumor biology underscores their potential as therapeutic targets. By helping connect mitochondria to motor proteins, MIRO1 enables mitochondrial trafficking and subsequent migration, invasion, and metastasis of cancer cells. Furthermore, use of MIRO1 reducer—a small molecule that promotes the proteasomal degradation of MIRO1 (71)—has been utilized *in vivo* to reduce squamous cell carcinoma tumor growth (21). However, there are several caveats to consider when targeting MIRO1. Targeting MIRO1 in neurons is likely to be highly toxic as MIRO1 is critical to neuronal development and prevention of neurodegeneration, with germline MIRO1 KO mice developing cyanosis and dying within 15 to 30 min after birth and neuronal-conditional MIRO1 KO leading to early onset of motor neuron disease (72). Additionally, MIRO1 is also known to have an anti-metastatic role in some cell types (65). On the other hand, MIRO2 may provide a novel therapeutic target in a multitude of cancer types. Underscoring this idea, MIRO2 is often upregulated in cancer compared to normal tissues (19), and MIRO2 is critical for PCa growth *in vitro* and *in vivo* (18). Unlike MIRO1, germline MIRO2 KO mice are born fully viable and fertile and without neurological disease, suggesting that loss of MIRO2 is well tolerated (72). This is likely due to normal mitochondria trafficking even in the complete absence of MIRO2 expression. Diving into some of these open areas will hopefully lead to some exciting discoveries on the function and regulation of MIROs and help serve as the basis for therapeutic targeting of these proteins.

## Conflict of interest

The authors declare no conflict of interest with the contents of this article.
